# Transforming growth factor-β stimulates nerve growth factor production in osteoarthritic synovium

**DOI:** 10.1186/s12891-019-2595-z

**Published:** 2019-05-10

**Authors:** Shotaro Takano, Kentaro Uchida, Makoto Itakura, Dai Iwase, Jun Aikawa, Gen Inoue, Manabu Mukai, Masayuki Miyagi, Kosuke Murata, Hiroyuki Sekiguchi, Masashi Takaso

**Affiliations:** 1 Department of Orthopedic Surgery, Kitasato University School of Medicine, 1-15-1 Minami-ku, Kitasato, Sagamihara City, Kanagawa 252-0374 Japan; 2 Department of Biochemistry, Kitasato University School of Medicine, 1-15-1 Minami-ku, Kitasato, Sagamihara City, Kanagawa 252-0374 Japan; 3 Shonan University of Medical Sciences Research Institute, Nishikubo 500, Chigasaki City, Kanagawa 253-0083 Japan

**Keywords:** Transforming growth factor-β, Nerve growth factor, Osteoarthritis, Synovium

## Abstract

**Background:**

Nerve growth factor (NGF) contributes to pain in knee osteoarthritis (KOA) patients. Transforming growth factor-beta (TGF-β) stimulates NGF expression in chondrocytes from KOA patients. However, the correlation between synovial TGF-β and NGF levels has not been sufficiently studied in human KOA patients. Further, the mechanism governing NGF regulation by TGF-β in synovial cells is unclear.

**Methods:**

During total knee arthroplasty, we extracted the synovial tissue (SYT) of 107 subjects with unilateral Kellgren/Lawrence grade 3–4 KOA confirmed by radiography. We examined the distribution of TGF-β and NGF using immunohistochemistry, and analyzed the relationship between *NGF* and *TGFB* mRNA levels. Cultured synovial cells extracted from SYT were exposed to culture medium (control), human recombinant TGF-β (rhTGF-β), rhTGF-β + ALK5 inhibitor SB505124, rhTGF-β + transforming growth factor activating kinase 1 (TAK1) inhibitor (5Z)-7-oxozeaenol, or rhTGF-β + p38 inhibitor SB203580 for 30 min, 6 h and 24 h. *NGF* mRNA expressed by the cultured cells and NGF protein levels in the cell supernatant were detected by real-time polymerase chain reaction (PCR) and enzyme-linked immunosorbent assay (ELISA), respectively. Phosphorylation of p38 was evaluated by western blotting.

**Results:**

*NGF* mRNA levels were positively correlated with those of *TGFB*. Cells expressing TGF-β and NGF protein were observed in the lining layer of SYT. TGF-β stimulated increased *NGF* mRNA expression and NGF protein production. The ALK5 inhibitor completely suppressed the TGF-β-mediated increase in *NGF* expression and NGF production in synovial cells. ALK5, TAK1 and p38 inhibitors inhibited the TGF-β-induced phosphorylation of p38, and TAK1 and p38 inhibitors partially inhibited the TGF-β-mediated increase in *NGF* expression and NGF production in synovial cells.

**Conclusion:**

TGF-β regulates NGF production via the TGF-β/ALK5 signaling pathway in osteoarthritic synovium. This effect may partially occur through inhibition of the TAK1/p38 pathway in the SYT of KOA patients.

**Electronic supplementary material:**

The online version of this article (10.1186/s12891-019-2595-z) contains supplementary material, which is available to authorized users.

## Background

Knee osteoarthritis (KOA) is a common form of joint disease and a leading cause of disability and pain. Its symptoms include pain and reduced mobility, which reduce patients’ quality of life [1]. Pharmacological treatments are generally aimed at relieving pain and improving joint function. However, therapies like nonsteroidal anti-inflammatory drugs (NSAIDs) have limited efficacy, and are associated with serious adverse effects including renal, cardiovascular, gastrointestinal, and cardiovascular complications [2]. Understanding the pathways that govern pain in KOA patients may lead to improved drug treatments.

Nerve growth factor (NGF) is a neurotrophin that modulates nociception. It is elevated in chronic pain conditions, leading to increased perception of pain [3]. A recent clinical trial showed that neutralizing NGF was more effective than placebo and NSAIDs for reducing KOA pain [4–7]. Numerous studies have shown that NGF is regulated by inflammatory cytokines, including tumor necrosis factor (TNF)-α and interleukin (IL)-1β, in mouse and human articular chondrocytes, synovial fibroblasts, and synovial macrophages in vitro [8–11]. Synovial tissue (SYT) is a major component of joint inflammation. Interestingly, less inflammation has been observed in the synovium of late stage than early stage OA patients [12]. NGF neutralization reduces pain in both inflammatory and non-inflammatory states [13]. Therefore, synovial NGF regulation under non- or moderate synovial inflammatory states may play an important role in OA, particularly in the late stages of the disease. However, synovial NGF regulation under non- or moderate-inflammatory states is not fully understood.

Several studies have reported that anti-inflammatory cytokines such as transforming growth factor (TGF)-β and IL-10 regulate NGF. Recombinant IL-10 stimulates NGF in astrocytes in vitro [14]. However, studies have found no evidence of an elevation in IL-10 in the synovial fluid or serum of OA patients [15, 16]. In contrast, several clinical studies have shown that the TGF-β is elevated in the synovial fluid of KOA patients [17, 18]. Moreover, a recent study showed that TGF-β stimulated *NGF* mRNA expression in osteoarthritic articular cartilage in vitro, suggesting that TGF-β may contribute to pain in non-inflammatory OA [19]. These findings suggest that TGF-β may be one of the key molecules involved in NGF regulation in OA. However, the relationship between NGF and TGF-β expression in the osteoarthritic synovium is not fully understood.

In the canonical TGF-β signaling pathway, signaling is started by the binding of three TGF-β isoforms to the type II receptor (TβRII), and the subsequent phosphorylation of the type I receptor (TβRI) [20]. The phosphorylated TβRI, typically ALK5, can then transduce the TGF-β signal intracellularly to phosphorylate R-Smads. In contrast, the non-canonical TGF-β pathway signals through a non-Smad pathway via TGF-β-activated kinase 1 (TAK1), a member of the mitogen-activated protein kinase (MAPK) kinase kinase (MAPKK-K) family, which activates p38 and Jun N-terminal kinase (JNK) and the p38 MAPK pathway. Previous studies have shown that TGF-β regulates *NGF* mRNA expression via both the canonical and non-canonical pathway [19, 21]. The ALK5 inhibitor SB505124 completely blocked TGF-β-mediated *NGF* mRNA expression and partially suppressed the action of the TAK1 inhibitor 5Z oxozeanol in osteoarthritic cartilage. However, the effect of the canonical and non-canonical TGF-β pathways on NGF production in the osteoarthritic synovium is not fully understood.

Here, we investigated the mechanism governing the regulation of NGF by TGF-β via the canonical and non-canonical pathways in osteoarthritic synovium.

## Methods

### Patients

Ethics approval was obtained from the Institutional Review Board (IRB) for Clinical Research and Treatment of Kitasato University (IRB approval number: B13–113). The study sample size was decided by power analysis, with α = 0.05 and power = 0.95, using G*POWER3. The analysis demonstrated that 107 SYT samples were required to identify a statistically significant correlation between *TGFB* and *NGF* mRNA expression.

SYT samples were extracted during total knee replacement surgery from 115 patients with KOA of unilateral Kellgren/Lawrence grades 3 (49/115) and 4 (66/115) confirmed by radiography. Among these, 25 were men and 90 were women. The mean ± standard error (SE) age and body mass index (BMI) was 73.2 ± 0.7 years and 26.1 ± 0.4 kg/m^2^, respectively. Informed consent to participate in this study was obtained from all patients the day prior to surgery.

SYT samples were extracted from the suprapatellar pouch of the operated knee of each patient and immediately transferred to liquid nitrogen before storage in a freezer at − 80 °C until use for RNA extraction. SYT samples extracted from 24 patients were reserved for cell culture, while the remaining samples were fixed in 4% paraformaldehyde phosphate-buffered solution (Fuji Photo Film Co., Tokyo, Japan) for 48 h for histology.

### Quantitative real time polymerase chain reaction (qRT-PCR)

Total RNA extraction from SYT and cultured SYT cells and cDNA synthesis were performed according to a previously described method [22]. The following PCR primer pair sequences were used for qRT-PCR: *TGFB* (product size: 91 bp), *TGFB*-Forward (5′-CGACTCGCCAGAGTGGTTAT-3′) and *TGFB*-Reverse (5′-GCTAAGGCGAAAGCCCTCAA-3′); *NGF* (product size: 74 bp), *NGF*-Forward (5′-CCCATCCCATCTTCCACAGG-3′) and *NGF*-Reverse (5′-GGTGGTCTTATCCCCAACCC-3′); *TNAFA* (product size: 118 bp), *TNFA-*Forward (5′-CTTCTGCCTGCTGCACTTTG-3′) and *TNFA*-Reverse (5′-GTCACTCGGGGTTCGAGAAG-3′); *IL1B* (product size: 153 bp), *IL1B*-Forward (5′-GTACCTGTCCTGCGTGTTGA-3′) and *IL1B*-reverse (5′-GGGAACTGGGCAGACTCAAA-3′); and *GAPDH* (product size: 223 bp), *GAPDH*-Forward (5′-TGTTGCCATCAATGACCCCTT-3′) and *GAPDH*-Reverse (5′-CTCCACGACGTACTCAGCG-3′). Relative *TGFB* and *NGF* expression was evaluated using CFX-96® (Bio-Rad, Richmond CA, USA). *TGFB* and *NGF* mRNA levels were normalized to those of the housekeeping gene, *GAPDH*.

### Immunohistochemistry

To determine the localization of TGF-β and NGF protein, paraformaldehyde-fixed SYT was embedded in paraffin and cut into 4-μm-thick sections using a microtome (*n* = 10). The sections were deparaffinized in the xylene substitute Neo-Clear (Merck KGaA, Darmstadt, Germany) for 1 h, and subsequently hydrated in a series of decreasing ethanol concentrations (100, 95, and 70%) before rinsing in distilled water. The sections were subjected to antigen retrieval by submerging in sodium citrate buffer (10 mM sodium citrate acid, 0.05% Tween 20, pH 6.0) at 98 °C for 45 min. The sections were cooled at room temperature (RT) and exposed to 3% hydrogen peroxide in methanol for 20 min at RT to block endogenous peroxidases. The sections were rinsed with phosphate-buffered saline (PBS; 3 times, 10 min each) and incubated with 10% goat serum (Nichirei, Tokyo, Japan) at RT, followed by mouse monoclonal primary antibody against TGF-β (cat no. 27969, Abcam, Cambridge, UK) or rabbit polyclonal primary antibody against NGF (cat no. ab6199, Abcam) for 1 h at RT. The sections were rinsed with PBS (3 times, 10 min each) and incubated with biotinylated anti-rabbit IgG (Nichirei) for 10 min at RT. The sections were again rinsed with PBS (3 times, 10 min each) and then incubated with horseradish peroxidase (HRP)-conjugated streptavidin for 10 min. After rinsing with PBS (3 times, 10 min each), the sections were reacted with 3,3′-diaminobenzidine, rinsed in water, then counterstained with Mayer’s hematoxylin and mounted in mounting medium. Negative control mouse and rabbit IgG antibodies were used as negative controls for the mouse monoclonal antibodies and rabbit polyclonal antibodies, respectively. No positive cells were observed in the negative control sections.

### Cell culture

SYT was digested with 30 mL of 1 mg/ml collagenase solution, and synovial cells were isolated and then cultured (1 × 10^5^ cells/cm^2^) in α-minimal essential media (MEM) and 10% fetal bovine serum in six-well plates. The medium was changed twice across a 7-day incubation. To identify the synovial cell populations in culture, the cells were incubated with fluorescein isothiocyanate (FITC)-conjugated anti-CD45 (pan leukocyte marker), allophycocyanin (APC)-conjugated anti-CD90 (synovial fibroblast marker), and phycoerythrin (PE)-conjugated anti-CD14 (macrophage marker) antibodies. The fibroblast (CD45-CD90+) and macrophage fractions (CD45 + CD14+) were examined using flow cytometry (FACSVerse™; BD Biosciences, San Jose, CA, USA). We used isotype controls to set the positive gate.

Subsequently, cells derived from 8 patients were exposed to culture medium (control), 10 ng/mL rhTGF-β, or 10 ng/mL rhTGF-β + 5 μM SB505124 for 30 min, 6 h or 24 h. Cells derived from another 8 patients were exposed to culture medium (control), 10 ng/mL human recombinant TGF-β (rhTGF-β), or 10 ng/mL rhTGF-β + 1 μM 5Z-7-oxozeaenol (5Z) for 30 min, 6 h or 24 h. Cells derived from the remaining 8 patients were exposed to culture medium (control), 10 ng/mL rhTGF-β, or 10 ng/mL rhTGF-β + 1 μM SB203580 for 30 min, 6 h or 24 h. After stimulation for 30 min, p38 phosphorylation was evaluated by western blotting to monitor the effect of inhibitors on the non-canonical pathway. After stimulation for 6 and 24 h, total RNA was extracted from the cells for real-time PCR analysis and the cell culture supernatant was analyzed for NGF protein levels using an enzyme linked immunosorbent assay (ELISA) kit (R&D Systems, Minneapolis, MN, USA).

### Western blotting

To determine the mechanism governing the regulation of NGF by TGF-β, p38 MAPK phosphorylation was examined using western blotting. After stimulation for 30 min as described above, cell lysates were prepared using sodium dodecyl sulfate (SDS) sample buffer. Cell lysates (5 μg) were separated using SDS-polyacrylamide gel electrophoresis before electrophoretic transfer to a polyvinylidene difluoride membrane in blotting buffer. For blocking, the membrane was incubated with 10% nonfat milk in Tris buffered saline containing 0.05% Tween 20 (TBS-T) for 60 min at RT. Subsequently, the membrane was incubated with rabbit polyclonal primary antibodies against p38 MAPK (cat no. 9212; Cell Signaling Technology Japan, Tokyo, Japan) or phospho-p-38 MAPK (Thr180/Tyr182) (cat no. 9211; Cell Signaling Technology Japan) for 1 h at RT, followed by HRP-conjugated goat anti-rabbit IgG antibody for 1 h at RT. The membrane was rinsed 3 times with TBS-T, and the proteins were visualized by enhanced chemiluminescence using the ImageQuant LAS-4000mini (Fuji Photo Film Co). Bands were analyzed by densitometry using NIH ImageJ software and normalized to p38 expression. Relative p-p38/p-38 expression was determined based on the expression in control samples.

### Statistical analysis

The relationship between the mRNA expression levels of *NGF* and *TGFB*, *TNFA*, and *IL1B* in SYT was analyzed using Spearman’s correlation coefficient. Differences between control- and rhTGF-β-treated cells were analyzed using Bonferroni’s multiple comparisons test. All statistical analyses were performed using Statistical Package for the Social Sciences (SPSS) software (version 25.0, IBM, NY, USA). *P* < 0.05 was used to indicate statistical significant for all analyses.

## Results

### Correlation between *NGF* and *TGFB* mRNA levels in the SYT of KOA patients

**Fig. 1:**
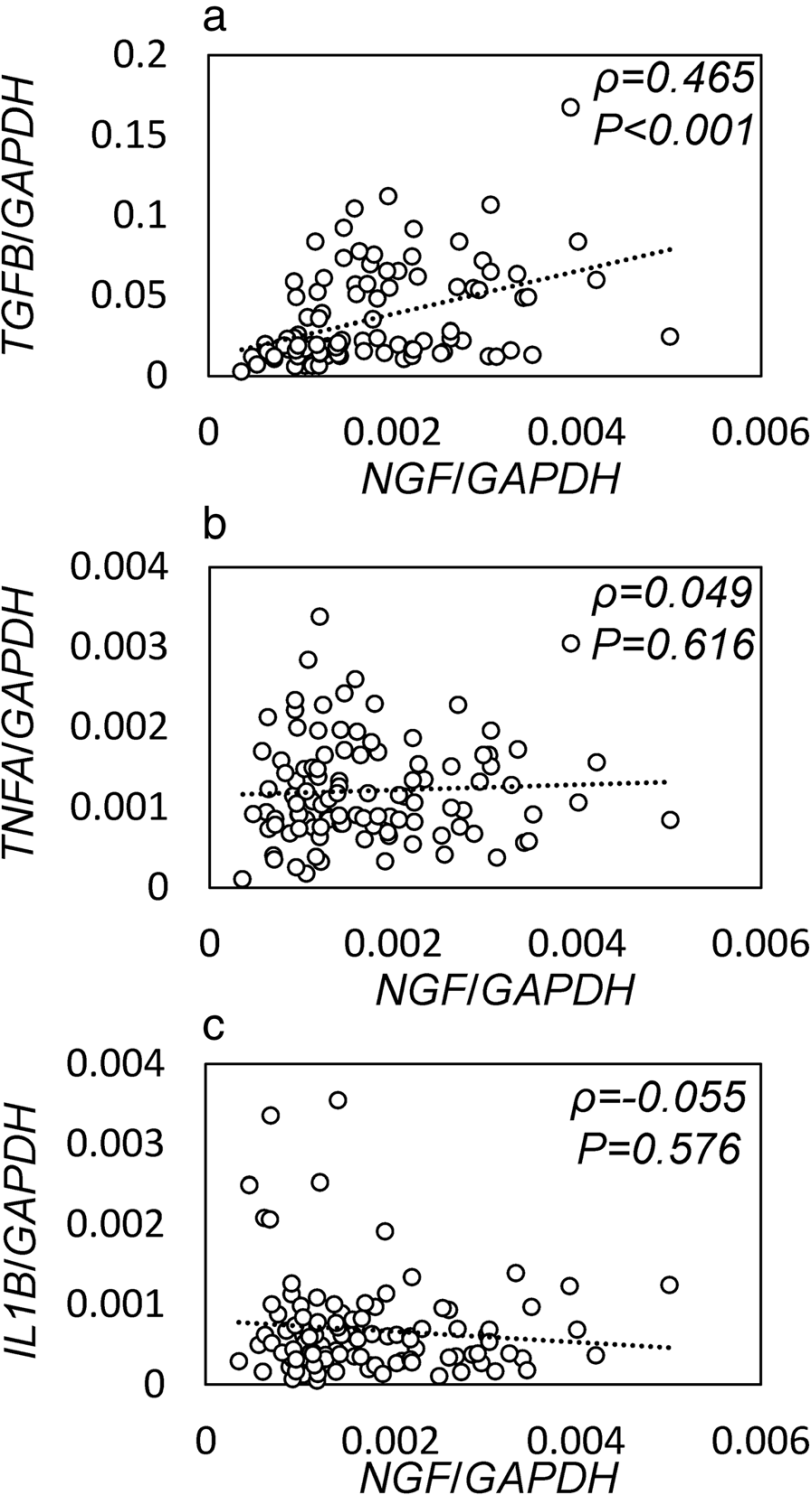
Correlation between the mRNA levels of *NGF* and *TGFB*, *TNFA*, and *IL1B* in synovial tissue.Relationship between *NGF* and *TGFB* (**a**), *TNFA* (**b**), and *IL1B* (**c**) in synovial tissue extracted from 107 patients with knee osteoarthritis

*NG*F mRNA levels were positively correlated with those of *TGFB* (ρ = 0.465, *P* < 0.001; Fig. 1a). In contrast, there was no correlation between *NGF* and *TNFA* or *IL1B* in the SYT (TNFA, ρ = 0.049, *P* = 0.616; IL1B, ρ = − 0.055, *P* = 0.576; Fig. 1b, c).

### Localization of NGF and TGF-β protein in the SYT of KOA patients

**Fig. 2:**
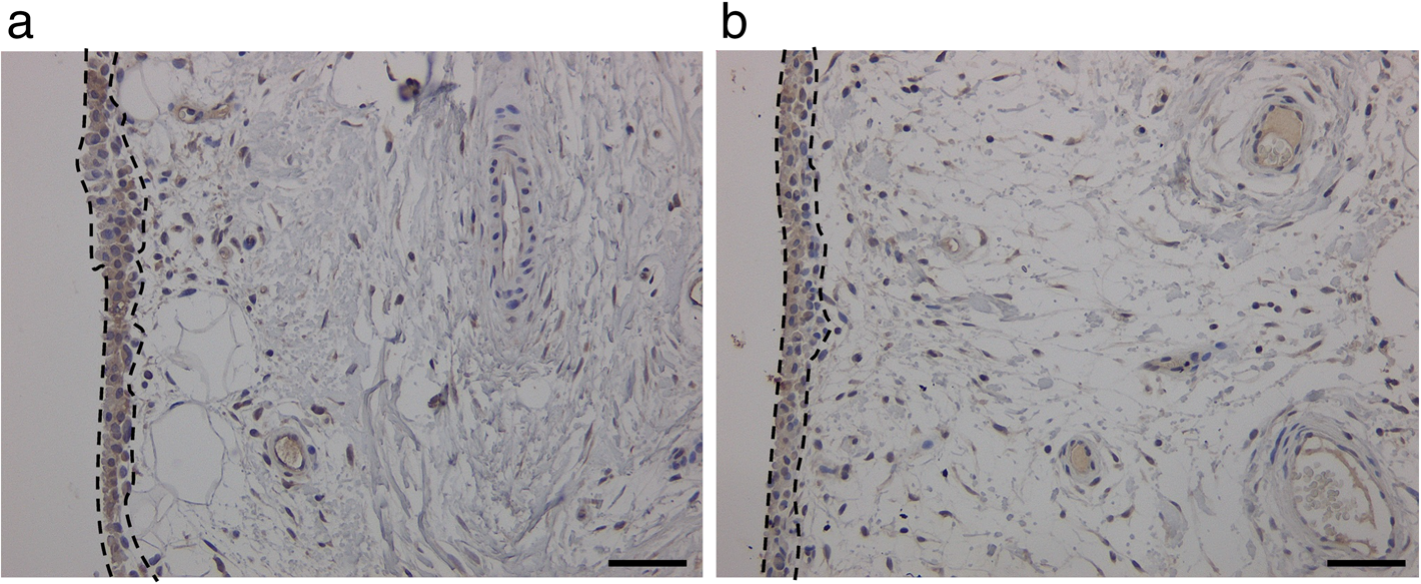
Immunolocalization of TGF-β and NGF in synovial tissue.Representative micrographs showing NGF (**a**) and TGF-β (**b**) protein expression (brown label) in the synovial lining layer. Region enclosed by dotted lines indicates the synovial lining layer. Scale bar = 100 μm

As qPCR analysis detected a correlation between *NGF* and *TGFB* mRNA expression, immunohistochemical analysis was performed to investigate the localization of NGF and TGF-β in SYT. NGF and TGF-β protein were detected in the lining layer of SYT (Fig. 2a, b).

### Effect of an ALK5 inhibitor on TGF-β-induced NGF expression and NGF production in synovial cells

**Fig. 3:**
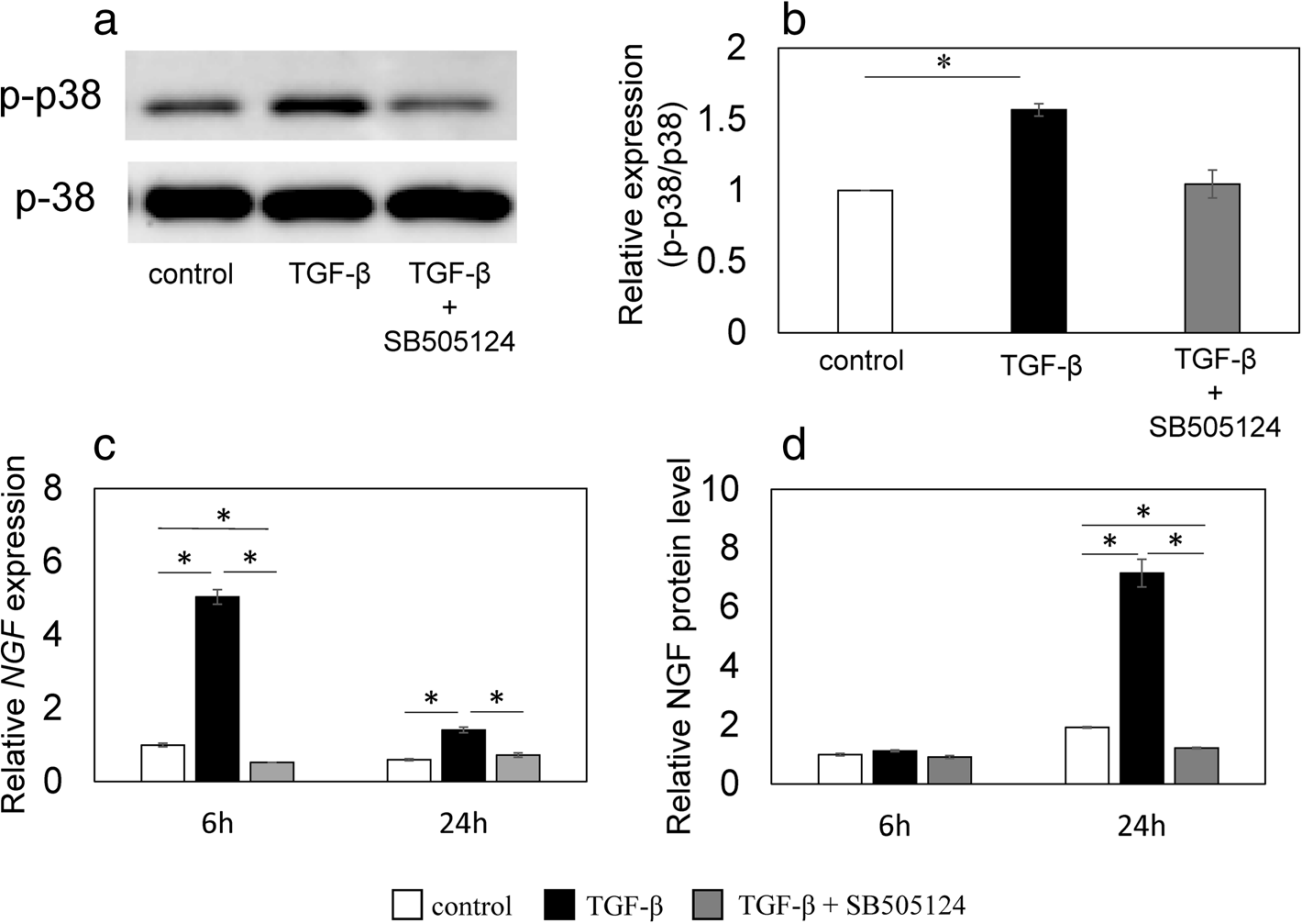
Effect of TGF-β and an ALK5 inhibitor on the phosphorylation of p38 MAPK, *NGF* mRNA expression, and NGF protein production. Western blotting for phosphorylated p38 MAPK (p-p38) (**a**-**b**). Synovial cells were exposed to α-MEM (control), 10 ng/mL human recombinant (rh) TGF-β (TGF-β), or 10 ng/mL rhTGF-β + 5 μM SB505124 (TGF-β + SB505124) for 30 min before protein extraction and western blotting. Western blot for p38 and phosphorylated p38 MAPK (p-p38) (**a**). Bands were analyzed by densitometry using the NIH ImageJ software and normalized to p38 expression. Relative p-p38/p-38 expression was determined based on the expression in control samples (**b**). RT-PCR (**c**) and ELISA (**d**) for NGF. Synovial cells were exposed to α-MEM (control), 10 ng/mL rhTGF-β (TGF-β), or 10 ng/mL rhTGF-β + 5 μM SB505124 (TGF-β + SB505124) for 6 and 24 h before RT-PCR (**c**) or ELISA (**d**). Relative expression was determined based on the expression in control samples. Data indicate mean ± SE (*n* = 8). *p < 0.05 compared to control. Data indicate mean ± SE (n = 8). *p < 0.05 compared to control

Flow cytometry demonstrated that cultured synovial cells were primarily made up of CD45-CD90+ fibroblasts (86.3 ± 0.5%, mean ± SE; Additional file 1: Figure S1A) and some CD45 + CD14+ macrophages (8.6 ± 0.6%; Additional file 1: Figure S1B). Stimulation with rhTGF-β increased p38 phosphorylation (*P* = 0.018; Fig. 3a, b) compared to control, and addition of 5 μM SB505124 decreased p38 phosphorylation back to control levels (Fig. 3a, b). Stimulation with exogenous rhTGF-β significantly elevated *NGF* mRNA levels after 6 and 24 h (*P* < 0.001 and P < 0.001, respectively; Fig. 3c), and addition of 5 μM SB505124 completely suppressed this elevation (P < 0.001 and P < 0.001, respectively; Fig. 3c). Stimulation with exogenous rhTGF-β elevated NGF protein concentrations in the supernatant after 24 h (*P* = 0.001; Fig. 3d), and 5 μM SB505124 completely suppressed this elevation (P = 0.001; Fig. 3d).

### Effect of a TAK1 inhibitor on TGF-β-induced *NGF* mRNA expression and NGF protein production in synovial cells

**Fig. 4:**
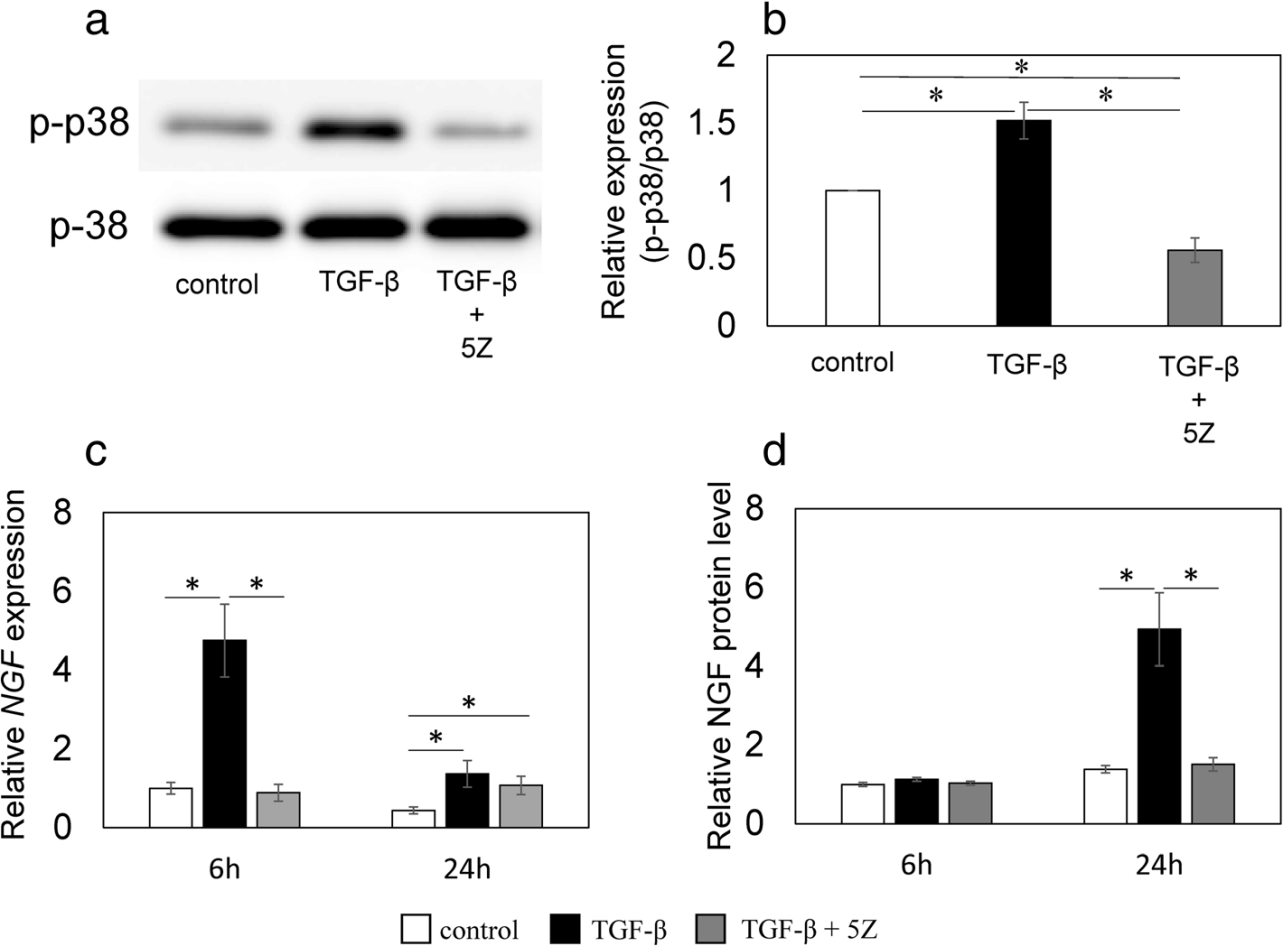
Effect of TGF-β and a TAK1 inhibitor on the phosphorylation of p38 MAPK, *NGF* mRNA expression, and NGF protein production. Western blotting for phosphorylated p38 MAPK (p-p38) (**a**, **b**). Synovial cells were exposed to α-MEM (control), 10 ng/mL human recombinant (rh) TGF-β (TGF-β), or 10 ng/mL rhTGF-β + 1 μM (5Z)-7-oxozeaenol (TGF-β + 5Z) for 30 min before protein extraction and western blotting. Western blot for p38 and phosphorylated p38 MAPK (p-p38) (**a**). Bands were analyzed by densitometry using the sNIH ImageJ software and were normalized to p38 expression. Relative p-p38/p-38 expression was determined based on the expression in control samples (**b**). RT-PCR (**c**) and ELISA (**d**) for NGF. Synovial cells were exposed to α-MEM (control), 10 ng/mL rhTGF-β (TGF-β), or 10 ng/mL rhTGF-β + 1 μM (5Z)-7-oxozeaenol (TGF-β + 5Z) for 6 and 24 h before RT-PCR (**c**) or ELISA (**d**). Relative expression was determined based on the expression in control samples. Data indicate mean ± SE (n = 8). *p < 0.05 compared to control. Data indicate mean ± SE (n = 8). *p < 0.05 compared to control

Stimulation with rhTGF-β increased p38 phosphorylation (*P* = 0.012; Fig. 4a, b) compared to control, and addition of 1 μM 5Z reduced this increase (*P* = 0.006; Fig. 4a, b). Stimulation with exogenous rhTGF-β significantly elevated *NGF* mRNA levels after 6 h (*P* = 0.002; Fig. 4c), and addition of 1 μM 5Z significantly reduced this elevation (P = 0.002; Fig. 4c). Stimulation with exogenous rhTGF-β also induced an increase in *NGF* mRNA expression at 24 h (*P* = 0.032; Fig. 4c), while the presence or absence of 1 μM 5Z had no effect on *NGF* mRNA levels (*P* = 0.107; Fig. 4c). Stimulation with exogenous rhTGF-β elevated NGF protein concentrations in the supernatant after 24 h (*P* = 0.016; Fig. 4d), and 1 μM 5Z significantly reduced this elevation (*P* = 0.017; Fig. 4d).

### Effect of a p38 inhibitor on TGF-β-induced *NGF* expression and NGF production in synovial cells

**Fig. 5:**
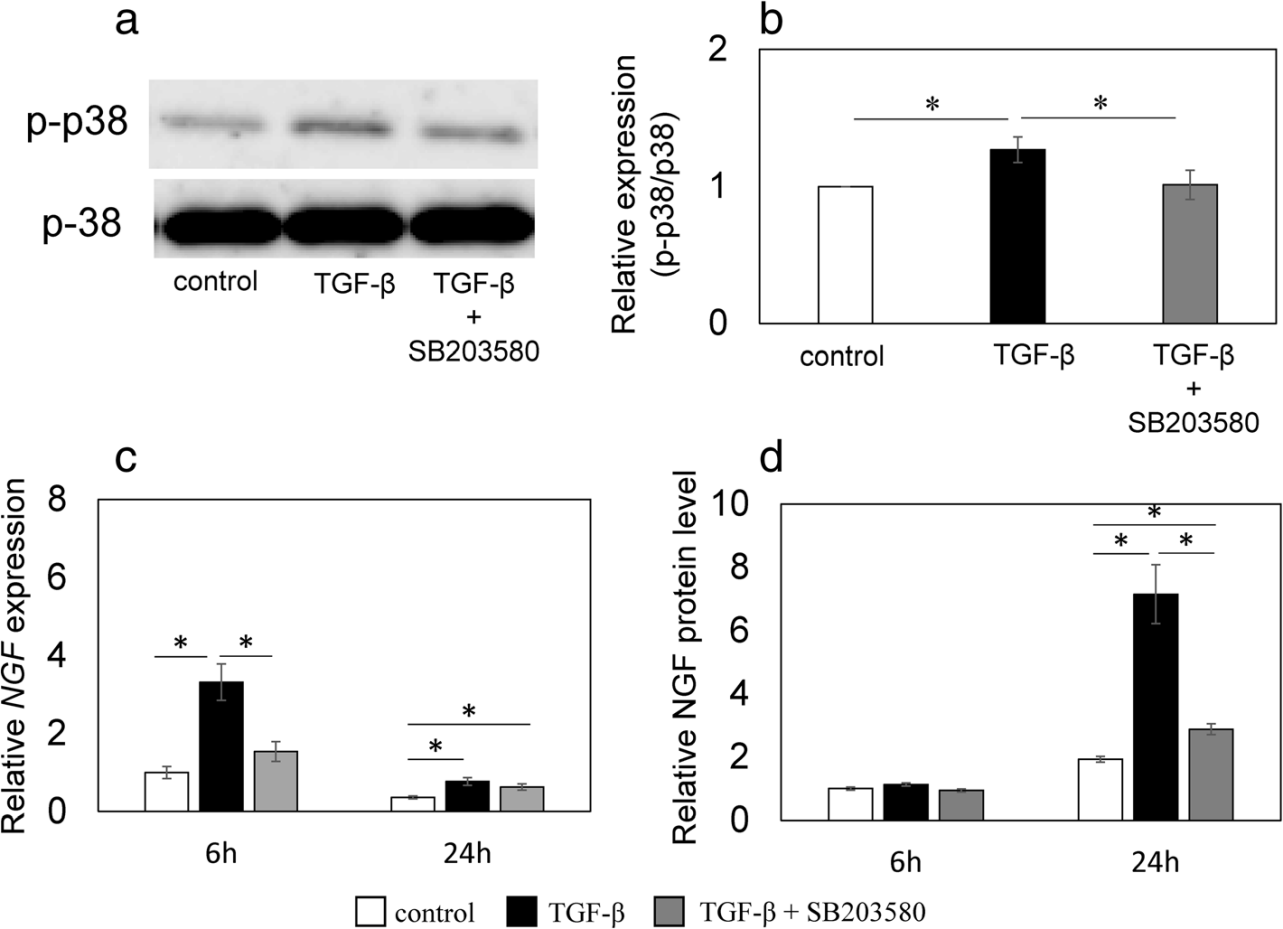
Effect of TGF-β and a p38 inhibitor on the phosphorylation of p38 MAPK Western blotting for phosphorylated p38 MAPK (p-p38) (**a**, **b**). Synovial cells were exposed to α-MEM (control), 10 ng/mL human recombinant (rh) TGF-β (TGF-β), or 10 ng/mL rhTGF-β + 1 μM SB203580 (TGF-β + SB203580) for 30 min before protein extraction and western blotting. Western blot for p38 and phosphorylated p38 MAPK (p-p38) (**a**). Bands were analyzed by densitometry using NIH ImageJ software and were normalized to p38 expression. Relative p-p38/p-38 expression was determined based on the expression in control samples (**b**). RT-PCR (**c**) and ELISA (**d**) for NGF. Synovial cells were exposed to α-MEM (control), 10 ng/mL rhTGF-β (TGF-β), or 10 ng/mL rhTGF-β + 1 μM SB203580 (TGF-β + SB203580) for 6 and 24 h before RT-PCR (**c**) or ELISA (**d**). Relative expression was determined based on the expression in control samples. Data indicate mean ± SE (n = 8). *p < 0.05 compared to control. Data indicate mean ± SE (n = 8). *p < 0.05 compared to control

The increase in rhTGF-β-mediated p38 phosphorylation (*P* = 0.045; Fig. 5a, b) compared to control was reduced by the presence of 1 μM SB203580 (Fig. 5a, b). Similarly, the exogenous rhTGF-β-mediated elevation in *NGF* mRNA expression after 6 h (*P* = 0.001; Fig. 5c) was significantly reduced by the presence of 1 μM SB203580 (*P* = 0.004; Fig. 5c). Further, the exogenous rhTGF-β-mediated elevation in NGF protein concentration in the supernatant after 24 h (*P* = 0.011; Fig. 5d) was significantly reduced by the presence of 1 μM SB203580 (*P* = 0.015; Fig. 5d).

## Discussion

We examined the mechanisms governing *NGF* regulation in the SYT of KOA patients. Our findings demonstrate a correlation between *TGFB* and *NGF* expression, and both TGF-β and NGF protein were localized to the synovial lining layer. The ALK5 inhibitor completely suppressed *NGF* mRNA expression, NGF protein expression, and p38 phosphorylation. Stimulation of synovial cells with rhTGF-β increased *NGF* mRNA expression and NGF protein production, which were partially inhibited by TAK1 and p38 inhibitors. Our results suggest that TGF-β/ALK5 signaling plays an important role in TGF-β-mediated NGF production and that its action may partially occur through inhibition of the TAK1/p38 pathway in the SYT of KOA patients.

Increased NGF levels have been reported in rheumatoid arthritis, spondyloarthritis, and osteoarthritis patients [23, 24]. Ours and other recent studies have demonstrated that stimulation with inflammatory cytokines such as TNF-α and IL-1β enhances NGF production from human and mouse synovial fibroblasts and macrophages in vitro [8, 10, 19, 25]. In the present study, analysis of human osteoarthritic SYT showed that NGF and TGF-β protein were localized to the synovial lining layer and that *NGF* mRNA levels were correlated with those of *TGFB* but not *IL1B* or *TNFA*. A previous study reported a significant increase in the number of TNF-α- and IL-1β-producing cells in the synovium of early stage OA patients undergoing arthroscopic surgery compared to the synovium of late stage KOA patients undergoing total knee arthroplasty (TKA) [12]. Our results, based on samples obtained from late stage KOA patients undergoing TKA, suggest that TGF-β may be one of the main regulators of NGF in the synovium of at least late stage KOA patients.

The canonical pathway plays a key role in regulating *NGF* mRNA expression. ALK5 inhibition suppressed TGF-β-induced *NGF* expression in a human pancreatic cell line and osteoarthritic chondrocytes [19, 21]. Consistent with these reports, we showed that an ALK5 inhibitor completely suppressed *NGF* mRNA expression and NGF protein production. These results suggest that the TGF-β/ALK5 pathway is important for NGF production in osteoarthritic synovium.

Several studies have suggested that non-canonical TGF-β pathway may mediate NGF expression via the MAPK pathway [19, 26]. TAK1 partially regulate TGF-β-induced NGF expression in human chondrocytes [19]. Phosphorylation of p38 MAPK, a downstream target of TAK1, is suggested to underlie the TGF-β-induced NGF expression in human dental pulp cells [26]. In contrast, previous studies have reported crosstalk between the canonical and non-canonical pathways [27, 28]. The ALK5 inhibitor SB505124 inhibits TGF-β-mediated p38 phosphorylation through suppression of the kinase activity of ALK4 and ALK5 [27]. In our study, we found that ALK5 inhibition with SB505124 suppressed TGF-β-mediated p38 phosphorylation in synovial cells. In addition, TGF-β promoted NGF production, which was partially inhibited by TAK1 and p38 inhibitors in synovial cells similar to previous report describing human cartilages [19]. This evidence suggests that TGF-β/ALK5 signaling-mediated NGF production may be partially regulated by the TAK1/p38 pathway in the SYT of KOA patients.

A number of randomized controlled trials have compared the efficacy and safety of tanezumab, a monoclonal antibody against NGF, with a placebo/active comparator for managing KOA pain [4, 6, 7]. Additionally, a recent meta-analysis showed that tanezumab was more effective for relieving pain and improving physical function and patient global assessment than placebo among KOA patients. Tanezumab is also generally well tolerated with minor adverse effects [29]. While we did not examine the relationship between *NGF* mRNA levels and pain in KOA patients, our findings regarding the regulation of NGF by TGF-β in SYT may be important for the development of future treatments for pain associated with KOA.

## Conclusions

TGF-β regulates NGF production via the TGF-β/ALK5 signaling pathway in osteoarthritic synovium. This effect may be partially due to inhibition of the TAK1/p38 pathway in the SYT of KOA patients. Our findings may be important for the development of future treatments for pain associated with KOA.

## Additional file

Additional file1: **Figure S1.** Flow cytometric analysis of cultured synovial cells. Dot-plot analysis of CD90 + CD45- (A) and CD14 + CD45+ (B) cells in synovial cell culture. (TIFF 870 kb)

## Abbreviations

APC: Allophycocyanin
BMI: Body mass index
FITC: Fluorescein isothiocyanate
IRB: Institutional review board
K/L: Kellgren/Lawrence
KOA: Knee osteoarthritis
NGF: Nerve growth factor
NSAIDs: Nonsteroidal anti-inflammatory drugs
OA: Osteoarthritis
PE: Phycoerythrin
SDS: Sodium dodecyl sulfate
SE: Standard error
SYT: Synovial tissue
TGF-β: Transforming growth factor-β
TβRI: TGF-β type I receptor
TβRII: TGF-β type II receptor
